# Beneath the floor: re-analysis of neurodevelopmental outcomes in untreated Hurler syndrome

**DOI:** 10.1186/s13023-018-0817-3

**Published:** 2018-05-11

**Authors:** Elsa G. Shapiro, Chester B. Whitley, Julie B. Eisengart

**Affiliations:** 1 Department of Pediatrics, University of Minnesota, Minneapolis, MN USA; 2 Shapiro Neuropsychology Consulting, LLC, Portland, OR USA; 3 Department of Experimental and Clinical Pharmacology, University of Minnesota, Minneapolis, USA

**Keywords:** Mucopolysaccharidosis type I, Developmental quotient, Neurodegenerative disease, Age equivalent, Cognitive decline, Newborn screening, Natural history

## Abstract

**Background:**

Hurler syndrome (MPS IH), the severe, neurodegenerative form of type one mucopolysaccharidosis, is associated with rapid neurocognitive decline during toddlerhood and multi-system dysfunction. It is now standardly treated with hematopoietic cell transplantation (HCT), which halts accumulating disease pathology and prevents early death. While norm-based data on developmental functioning in untreated children have previously demonstrated neurocognitive decline, advances in methodology for understanding the cognitive functioning of children with neurodegenerative diseases have highlighted that the previous choice of scores to report results was not ideal. Specifically, the lowest possible norm-based score is 50, which obscures the complete range of cognitive functioning at more advanced stages of neurodeterioration. To a set of cognitive data collected on a sample of untreated children, we applied a modern method of score analysis, calculating a developmental quotient based on age equivalent scores, to reveal the full range of cognitive functioning beneath this cutoff of 50, uncovering new information about the rapidity of decline and the profound impairment in these children.

**Results:**

Among 39 observations for 32 patients with untreated Hurler syndrome, the full array of cognitive functioning below 50 includes many children in the severely to profoundly impaired range. The loss of skills per time unit was 14 points between age 1 and 2. There was a very large range of developmental quotients corresponding to the norm-based cutoff of 50.

**Conclusions:**

This report enables clarification of functioning at levels that extend beneath the floor of 50 in previous work. At the dawn of newborn screening and amidst a proliferation of new therapies for MPS I, these data can provide crucial benchmark information for developing treatments, particularly for areas of the world where transplant may not be available.

## Background

Hurler syndrome, the severe, neurodegenerative form of type one mucopolysaccharidosis (MPS I), is now standardly treated with hematopoietic cell transplantation (HCT), which halts accumulating disease pathology and prevents early death. However, the vast majority of affected children are left with less than normal cognitive and adaptive skills [1–4] which interferes with their long-term quality of life. Earlier intervention owing to newborn screening, as well as a surge in innovative treatments, may allow better cognitive and adaptive development [5–7]. Yet comprehensive cognitive information about the untreated natural history is lacking. We have previously published norm-based data on untreated children from our center [1, 8, 9] but advances in methodology for understanding the cognitive functioning of children with MPS IH have highlighted that the previous choice of scores used to report results was not ideal. Specifically, the first and second editions of the Bayley Scales of Infant Development (BSID) [10, 11] that were used with these children had a lowest possible score of 50 (i.e., “floor”) and thus the lowest end of the developmental trajectory was necessarily truncated at that level: It did not reveal the full range of how profoundly impaired children may be, thus obscuring the complete natural history of this disorder [12]. Furthermore, it was not until the results of early HCT were published in 1996 and 1998 [13, 14] that age equivalent scores were first used, and since then, this methodology has become the gold standard for demonstrating cognitive functioning in highly impaired patients [3, 7, 9, 15–19]. However, these valuable data were never published for those children who were not transplanted, such that the literature is lacking detailed information about declining cognitive age equivalent scores and developmental quotients related to age.

This paper presents detailed cognitive information on untreated children with MPS IH, diagnosed before age 3 years. Most of them were part of an NIH study (NS 29099) from 1991 to 1997 exploring the effectiveness of bone marrow transplant (BMT) treatment. Because initial treatment with BMT was only possible with sibling donors, the majority of these children lacked available donors and were not transplanted. That changed when unrelated donors and cord blood (now called hematopoietic cell transplant, HCT, referring to all sources of donor cells) became part of the therapeutic armamentarium, enabling treatment of nearly all patients. Further, emergence of enzyme replacement therapy has enabled treatment, albeit inferior to HCT, for all children with MPS IH, even in parts of the world where there is insufficient access to transplant [20–23]. As such, assessment of completely untreated children is essentially impossible in the modern era; therefore, the current study is not repeatable but offers crucial information about cognitive age equivalents, described comprehensively.

## Methods

### Participants

Children who were diagnosed with MPS IH, mostly by urinary glycosaminoglycan (GAG) and enzyme determination, were sent to the University of Minnesota for consideration for BMT. This study comprises patients seen between 1985 and 1995. None were genotyped as this was previous to the era where this was standard procedure. All patients were seen by one of the authors (CW and/or WK^1^) and referred for developmental examination.^2^ All patients completed a consent form as required by the IRB. All patients in this sample are now deceased.

Most of the patients were untreated due to lack of available donors in the pre-ERT era (Group 1, *N* = 23), and a smaller proportion eventually proceeded to transplant but died, such that all data gathered represent their untreated status (Group 2, *N* = 9). Due to likely differences in health status between the two groups, a conservative analytic approach was adopted to provide additional perspective on the variability in this cohort, in which the entire group was analyzed and Group 1 and Group 2 were also examined separately. Two patients from this time period who did not have BMT were eliminated because they were likely patients with attenuated MPS I (Hurler-Scheie syndrome), as they were diagnosed later and were more cognitively intact. Furthermore, as we have no follow-up, we have no indication that either are deceased.

### Procedures

Children who were seen prior to 1993 were administered the original BSID [10], and those after 1993 were administered the second edition [11]. While both the Mental and Physical scales of the BSID were administered in most cases, the Mental Scale results are reported here. We report 1) age at testing; 2) year of testing; 3) age equivalent score for the mental scale; 4) developmental quotient (DQ; defined as mental age divided by chronological age times 100 [15]); 5) MDI (standardized score of cognitive development) for comparison to developmental quotient; and 6) age at death. Information regarding vital status was gathered from two sources 1) communication to WK who stayed in contact with many of these patients; and 2) if we did not have the exact date, internet sources such as social security death index.

## Results

Findings are summarized by group in Table 1. All patient-level results are presented in Table 2. There were 32 patients who had at least one visit, did not have BMT at the time of visit, and had a BSID I or II. Seven patients had 2 visits; thus a total of 39 observations were made.

**Table 1:** Summary data for the full sample and each group separately. Ages are in months unless otherwise indicated

	Age at test	Mental age	DQ	SS/MDI	Age at death (Years)
Group 1 (*N* = 23)					
Mean	27.00	15.06	61.04	63.93	7.41
SD	16.60	5.76	21.89	17.55	2.81
Median	22.80	16.80	58.90	56.00	6.90 range: 3.3–11.47
Group 2 (*N* = 9)					
Mean	21.41	16.75	83.33	82.00	3.38
SD	10.80	5.70	12.92	15.43	2.35
Median	18.50	14.60	85.00	82.00	1.85 range: 1.22–7.71
Total Sample (*N* = 32)					
Mean	25.19	15.61	68.29	69.80	6.24
SD	15.05	5.73	21.97	18.76	3.23
Median	21.40	15.70	70.30	66.50	5.85 range: 1.22–11.47

Group 1 = Untreated

Group 2 = Eventually proceeded to BMT but died

Age at Test = age at time of neurocognitive testing

Mental Age = BSID Age Equivalent Score

DQ = Per Delaney et al. [15], Mental Age divided by Age at Test, times 100

SS/MDI = norm-based Bayley standard score, where mean = 100, SD = 15, and floor is 50

**Table 2:** Raw data for all patients. Ages are presented in months unless otherwise specified

ID	HCT?	Sex	Year seen	Visit #	Bayley edition	Age at test	Mental age	DQ	SS/MDI	Age at death (Years)
H201	No	female	1991	1	1	13.8	12.5	90.4	90	11.13
			1992	2	1	22.8	14.0	61.5	50	
H203	No	male	1990	1	1	7.1	2.2	31.0	50	8.17
H205	No	male	1992	1	1	21.9	16.8	76.8	64	8.74
H207	No	male	1992	1	1	13.3	12.5	93.9	100	N.A.
H210	No	male	1990	1	1	42.7	19.0	44.5	67	10.48
H217	No	female	1995	1	2	40.4	20.6	50.9	50	N.A.
			1995	2	2	41.1	19.7	48.0	50	
H218	No	male	1989	1	1	12.8	11.3	88.5	82	7.12
H220	No	male	1990	1	1	36.8	24.4	66.4	50	11.00
H223	No	male	1992	1	1	17.3	14.2	81.9	78	6.21
H225	No	female	1992	1	1	25.7	20.0	77.7	81	11.47
H226	No	male	1991	1	1	11.0	7.1	64.5	56	4.12
			1992	2	1	14.2	7.0	49.2	50	
H228	No	female	1994	1	2	36.2	27.0	74.6	74	13.36
H232	No	female	1988	1	1	30.2	17.8	58.9	50	6.68
H238	No	male	1987	1	1	91.9	12.2	13.3	50	8.77
H239	No	female	1988	1	1	22.0	17.0	77.4	68	5.13
H240	No	male	1990	1	1	18.8	10.0	53.3	50	4.05
H247	No	female	1987	1	1	36.1	19.3	53.5	50	5.85
H249	No	female	1991	1	1	80.5	17.8	22.1	50	4.67
H291	No	female	1985	1	1	32.1	14.0	43.7	50	4.81
			1985	2	1	35.3	17.8	50.4	50	
H328	No	female	1994	1	2	16.9	17.0	100.6	103	8.97
H341	No	male	1994	1	2	16.8	7.6	45.2	56	3.30
H357	No	male	1995	1	2	24.5	20.0	81.5	94	N.A.
H375	No	female	1995	1	2	16.1	7.8	48.4	63	8.14
H227	Yes	female	1994	1	2	15.8	14.6	92.4	94	7.71
			1994	2	2	20.9	17.8	85.0	85	
H234	Yes	male	1988	1	1	26.8	19.3	71.9	66	6.38
H243	Yes	male	1989	1	1	14.4	12.0	83.3	82	1.22
H244	Yes	female	1993	1	1	20.4	17.6	86.3	81	1.64
H292	Yes	male	1986	1	1	11.0	11.8	107.3	106	3.70
H297	Yes	female	1989	1	1	15.2	14.2	93.2	98	1.83
H299	Yes	male	1993	1	1	13.5	12.4	91.9	97	1.72
H302	Yes	female	1988	1	1	25.6	17.6	68.7	57	4.38
			1988	2	1	32.7	20.6	63.1	63	
H336	Yes	male	1992	1	1	12.9	12.2	94.4	94	1.85
			1992	2	1	18.5	14.6	78.8	76	

Age at Test = age at time of neurocognitive testing

Mental Age = Bayley Age Equivalent Score

DQ = Per Delaney et al. [15], Mental Age divided by Age at Test, times 100

SS/MDI = norm-based Bayley standard score, where mean = 100, SD = 15, and floor is 50

**Fig. 1:**
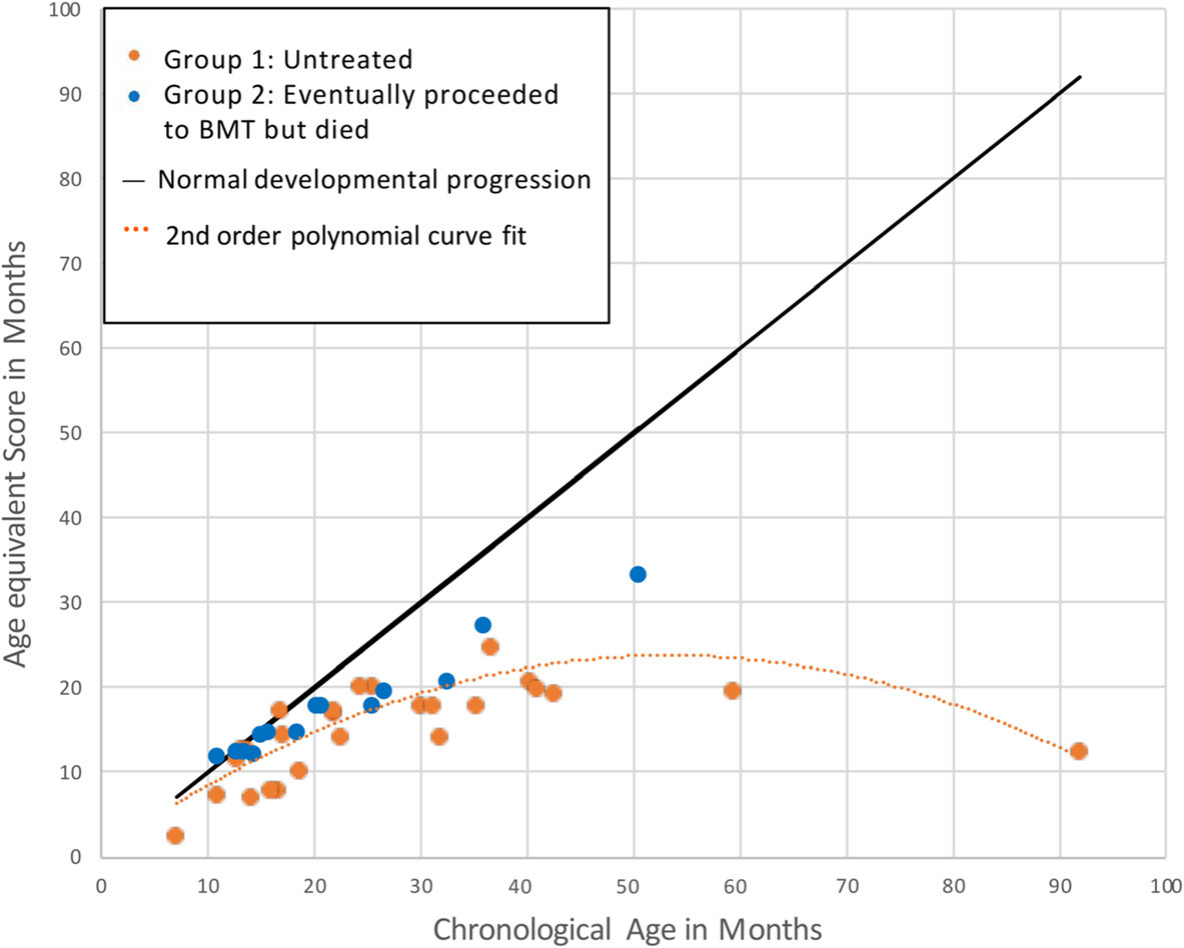
Developmental Growth Curves for Untreated Hurler Syndrome; Seen between 1983 and 1995. Developmental growth curves depict the patients’ mental ages as compared with their chronological ages at the time of testing. At younger chronological ages, most patients are measuring closer to the curve of normal development. With age, their mental functions depart from the normal developmental trajectory, revealing a plateauing in development and eventual decline, illustrated with a second order polynomial curve fit to the data

The developmental growth curve in Fig. 1 elucidates the early growth and plateauing in this group, with evidence of very low functioning after three years of age.

The mean DQ for patients seen between 1 and 2 years of age was 78.1 (S.D. = 17.7, *n* = 19); between 2 and 3 years of age was 64.5(13.1, 8); and between 3 and 4 years was 56.3 (19.4, 6). Thus, the change per year respectively was 14 and 8 DQ points. Median DQs were similar (83, 66, and 52 respectively) with a change per year of 17 and 14 points respectively.

For those patients who had an MDI over 50, the correlation with DQ was 0.87. For those who had an MDI of 50 or less (*n* = 13), the range of DQs was 13.3 to 66.4.

**Fig. 2:**
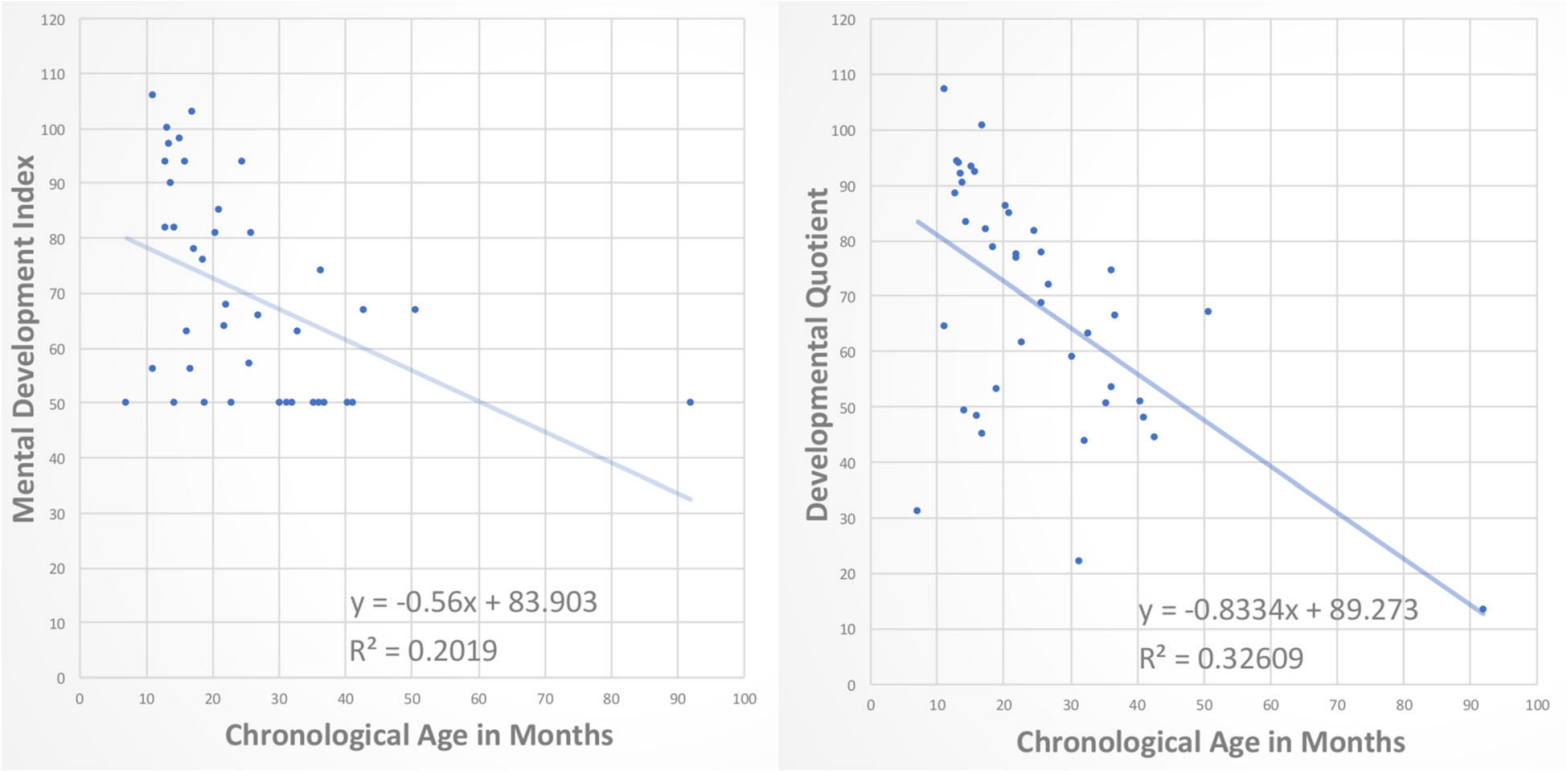
Comparison of slopes of decline using Standard Score versus Developmental Quotient (DQ). When plotted against chronological age, Standard Scores reveal the floor effect, with many data points clustered at a score of 50 (left), which affects calculation of slope of decline. By contrast, use of DQ scores eliminates the floor effect and shows many data points lower than 50 (right). With more comprehensive representation of DQs, the slope of decline is more accurately calculated as steeper

Figure 2 compares the MDI to the developmental quotients for all 32 patients; the slope of cognitive decline is steeper for the DQ than for the MDI for the same patients.

## Discussion

This study presents the natural history of children with MPS IH in using modern analytic methods, to provide detailed clarification of the trajectory of neurocognitive decline using age equivalent scores. In the modern era, several works have characterized neurocognitive functioning in MPS IH using age equivalent scores [2, 3], particularly to shed light on treatment outcomes, yet none have quantified the loss in developmental quotient points per year on an untreated population. Previous reports of this decline have used standard scores, which highlights the limitation of past methodology. A graph in Krivit, Peters & Shapiro [8] shows the floor of 50 on the BSID mental scale, and some of these patients overlap with our patients depicted in Fig. 2. Common to both the original graph [8] and the current graph depicting the Mental Development Index (MDI) in Fig. 2, children falling below this floor of 50 could not be sensitively monitored. This contrasts with the present study’s new graph in Fig. 2, depicting the developmental quotient of these same children, where many data points are beneath the floor.

Unlike the historical graph [8], we did not include patients who were transplanted after 1995 or those who survived in our new analyses, in order to focus on the untreated natural history. This cohort comprises patients who lacked available donors (Group 1) as well as patients who eventually proceeded to BMT but died (Group 2), such that all cognitive data represent the untreated natural history. The groups were believed possibly to be different, which is reflected in higher scores in Group 2, for a few reasons. First, for those who did not have an HLA identical sibling, the search for a matched donor may have delayed the assessments, leading to accumulating disease burden, including lower DQ scores and poorer health status in Group 1. Second, the mean age at testing was younger in Group 2, which may indicate less accumulated disease effects. However, the variability is greater in Group 1 than Group 2, while the medians are closer together, which assures some overlap of ages in the two groups. Last, physicians may have been more reluctant to perform a BMT on a patient with more cognitive impairment. As these patients lived in an era when comparatively less was known about MPS IH, much less data on health status, functioning, and treatment planning were routinely collected in standard fashion. Thus, these explanations for differences are speculated based on current knowledge.

This report uncovers the degree of neurodeterioration that extends beneath the floor of 50 in previous work. The full array of cognitive functioning in untreated MPS IH includes many children in the severely to profoundly impaired range, below 50 (33%) with a preponderance of those over 30 months of age (9 of 13). This cohort displayed a loss of skills per time unit (14 points in the 1–2 year olds) similar to that of Sanfilippo syndrome type A, another neurodegenerative type of MPS (i.e., MPS III A) [16, 24] with a decline of 14.6 points per year. Because of the few MPS IH patients over age 4 the decline cannot be as clearly defined beyond that chronological age. Similar to MPS III A patients who do not exceed an age equivalent score much over 30 months [16, 24], the highest age equivalent attained in this sample was 24.4 months.

There is a good correspondence between the developmental quotients and the standard scores in those with an MDI over 50. However, the 13 children with a 50 MDI had a very large range of DQ scores from 13.3 to 66.4, highlighting the arguments of previous work that standard scores do not sensitively reflect the true status of cognitive functioning in neurodegenerative disorders of childhood [15].

Raw scores, i.e., the number of items correct or points earned on the test, would have been another way to present these data; however, raw scores are less meaningful because they differ depending on the edition of the test or the age bracket of the child, and therefore fewer points could simply reflect a young age or different test. Age equivalent scores are the easiest and most appropriate approach to level the artificial differences. Delaney et al. [15] note that in severely impaired children, age equivalent scores enable monitoring of children with cognitive decline to clarify the effects of the disease process prospectively on the gaining of milestones, halting in developmental progress, and losing skills already acquired. A consensus conference on cognitive endpoints in clinical trials in MPS disorders also recommended the use of age equivalent scores to track the inflection points in the developmental curve of developmental arrest and subsequent decline [18]. The sensitivity of this approach has been demonstrated in MPS III [16].

The age of this data set is the most serious limitation. Medical science has progressed and particularly with the use of enzyme replacement [22, 23], survival is much better than it was during the time period that this data set was collected. In addition, this data set may not be fully representative of modern patients with MPS IH. First, cognitive impairment may have been more severe in this data set than currently for several reasons. Specifically, diagnoses were not made as early, as physicians were less informed about rare diseases and diagnoses were often delayed. Further, cognitive impairment can be tied to somatic difficulties which are now better managed [22, 25, 26]. Second, given that these children were all seen before the era of genotyping, the lack of information on the array of MPS IH genotypes represented may limit the generalizability. Third, this data set covers a narrow age range, with few patients over age 42 months, which limits characterization of cognitive status at older ages. It is speculated that accumulating disease burden limited travel, or that families may not have been motivated to pursue additional neurocognitive assessments in the absence of any feasible treatment options. Last, it should be noted that most of these children were seen at the University of Minnesota. Initially it was the first center in the United States to offer transplant as well as to provide opinions regarding its feasibility. Nevertheless, these are the most unalloyed data available on the cognitive course of untreated MPS IH. Historical data offer a unique perspective on the natural history of MPS IH, and may be closer to an authentic demonstration of the raw, untreated course of the disease, than can be obtained with current data on untreated children. That is, current diagnostic (e.g., MRI, SSEPs), interventional (e.g., shunting, antibiotics, hearing aids), supportive (e.g., physical therapy, speech/language) and palliative techniques are now more sophisticated and better tailored to the needs of people with MPS IH, which likely leads to better functioning even in children who do not have access to transplant, than this historical cohort.

The present study aims to increase understanding of the rate of neurocognitive decline in untreated MPS IH, which is needed for comparison when measuring the efficacy of therapies. Due to strong evidence that earlier intervention leads to more favorable outcomes in MPS I [1–3, 6], it was added to the Recommended Uniform Screening Panel (RUSP) for newborn screening. However, it is accepted that currently approved therapies are not perfect, and that people with MPS IH experience significant persisting disease burden [1, 2, 20, 23, 27–29]. This foundation of research and the addition of MPS I to the RUSP have energized the proliferation of new therapies for MPS I, many of which are currently in trial. The loss of DQ points quantified in the present study, and the refined calculation of the slope of decline, shows that neurodeterioration in MPS IH is even steeper and more devastating than previously understood. With the potential for earlier treatment increasing with newborn screening, the present study further argues for expeditious therapeutic intervention, ideally in the first few months of life.

## Conclusions

The natural history of MPS IH involves deterioration to profound intellectual impairment, which we have been able to quantify for the first time by using modern analytic methods to revisit data that were previously obscured by a norm-based cutoff of 50. Due to the array of therapies currently available as well as those on the horizon, this study will never be repeatable yet offers crucial enhanced understanding of the untreated course of this disease. Therefore, at the dawn of newborn screening and amidst a proliferation of new therapies for MPS I, these data can provide important benchmark information for developing treatments particularly for areas of the world where transplant may not be available.

## Abbreviations

DQ: Developmental quotient
GAG: Glycosaminoglycan
HCT: Hematopoietic cell transplantation
MPS I: Mucopolysaccharidosis type I
MPS IIIA: Mucopolysaccharidosis type IIIA, Sanfilippo syndrome

## Footnotes

1 William Krivit, MD PhD; Deceased, December 8, 2005. Principal investigator of the NIH grant (#NS 29099) that recruited many of these patients; he and Dr. Whitley screened for and performed the initial bone marrow transplants at the University of Minnesota.

2 Either by author (ES) or by Pi-Nian Chang, Ph.D., now retired.
